# Chronological approach to the characteristics of Ecuador’s debt with the IMF and the WB.

**DOI:** 10.12688/f1000research.154378.1

**Published:** 2024-08-23

**Authors:** Martha Lucía Romero Flores, Marcelo Sánchez-Oro Sánchez, Patricia Alexandra Chiriboga Zamora, Ligia Ximena Tapia Hermida

**Affiliations:** 1 Dirección de Investigación, Universidad Nacional de Chimborazo, Riobamba, Chimborazo Province, Ecuador; 2 Dirección de Doctorado en el programa de Desarrollo Territorial Sostenible, Universidad de Extremadura, Badajoz, Extremadura, Spain; 3 Business Administration Career, Universidad Nacional de Chimborazo, Riobamba, Ecuador

**Keywords:** External debt, World Bank, International Monetary Fund, development

## Abstract

Where did Ecuador’s foreign debt originate and grow to alarming levels? The purpose of this article is to describe, from an analytical perspective based on the contributions of various authors, the origin of Ecuador’s external debt with international institutions such as the World Bank and the International Monetary Fund, as well as the support that these organizations provide for the development of the countries of the South, case Ecuador. To achieve this purpose, a documentary methodology was implemented that included an exhaustive review of studies focused on the specific variables subject to analysis, the PRISMA tool and Atlas.ti 23 were used. The results show that the creation of these financing agencies in their attempt to support the financial stability of developing countries has not been sufficiently effective in improving the welfare conditions of a good percentage of the population and has generated an imbalance in the sustained development of the poorest countries. It is concluded that the capitalist system gives predominant priority to the economic growth of nations, thus relegating in its model the consideration of human development. This orientation does not contribute effectively to the improvement of crucial aspects such: education, health and the standard of living of the population.

## 1. Introduction

Financial institutions play an essential role in fostering economic growth and development by strengthening investment and promoting advances in productive technology and trade within a given economic system. However, despite the existence of policies designed to improve a country’s domestic conditions, they sometimes fail to do so. Ecuador continues to depend on international financial institutions to balance its balance of payments and is therefore subject to their conditions, which has given rise to significant difficulties, especially in the political and economic spheres.

Since its establishment as a republic, Ecuador has inherited a debt burden that has experienced alarming growth over time. Consequently, it is imperative to scrutinize the process of conception and granting of this financial support throughout its history, in order to clarify the underlying reasons why the policies implemented by the different governments have failed to consolidate the country’s economic development. It is also of utmost importance to carry out an exhaustive investigation with the objective of understanding the causes that have given rise to an unequal distribution of income in the various strata of Ecuadorian society, in spite of the abundant natural wealth of the nation.

It is essential to understand the political, economic and social conditions that have prevailed during the different governmental periods, as well as to evaluate the progress of key indicators such as poverty, inequality, health, employment, education and production, key factors in the measurement of human development.

With the documents studied, it is concluded that the policies implemented by these organizations have reduced the opportunities for growth towards development, it is considered that the financial organizations act in a restrictive manner, imposing on debtor countries the adoption of internal policies that harm the most vulnerable sectors, intensifying inequality and worsening poverty indexes. The current political and economic crisis in Ecuador, devastated by drug trafficking, corruption and crime, makes it highly vulnerable to a financial meltdown, with the consequent need for external financing and therefore absolute dependence on the conditions of its financiers.

## 2. Methods

Descriptive documentary research, using the PRISMA methodology (Preferred Reporting Items for Systematic reviews and Meta-Analyses), in which the actors who have carried out studies on the bibliographic bases of Science direct, Scopus, Pub med and Google scholar and the repository of the Central University of Ecuador were identified. In this way, the number of coincidences with respect to the variables under study was determined and then, through elimination, the articles that analyze the concepts of development, international financing organizations IMF and WB and the application of these organizations in Ecuador were determined. Illustration 1-2, based on Atlas’ti, shows the weight of the concepts discussed in the bibliographic databases consulted.

The systematic matrix was used to study the authors’ coincidences in previous works on the variables by analyzing the findings. Identified records:

Databases (n =3.998) Records marked as eligible (n=1.187) Records deleted for other reasons (n= 2.811). Records reviewed (n =60) Excluded records (n = 5). Reports sought for retrieval (n = 31) Unrecovered reports (n =5). Reports evaluated for eligibility (n =60). Studies included in the review (n = 55).

## 3. Results

### 3.1 Background

**3.1.1 Agricultural production**

The beginning of Ecuador as a republic dates back to 1809, when it was part of Gran Colombia and fought for independence from the Spanish colony. At that time, it was necessary to strengthen its armament and increase the number of soldiers to face the hard battles. In 1818 the Colombian and Venezuelan commissioners sought resources in Londres to finance the legitimate battle and in 1821 they signed the first contract for about £.186,475 (pounds sterling). In 1822 the loan of 2 million pounds sterling was acquired, and in 1824, £. 4.750.000, amortizing £.124.050, receiving a total of £.6.625.950, values destined to the acquisition of war armament, which later were branded as an overpriced purchase and for which, in spite of the disagreements of the assembly, it was agreed to pay ( Paz y Miño, 2021). The responsibility for the beginning of a debt that to this day has not allowed a positive balance lies in the poor conditions of purchase and the prospects of the negotiators. In 1830 Ecuador separated from the great Colombia, having inherited the co-responsibility of a debt distributed at the discretion of the managers of Colombia and Venezuela, with 21.5% corresponding to Ecuador, giving rise to a totally inequitable.

As expressed,

Alfaro (1896) “Venezuela and New Granada settled their foreign debt in a reasonable and honest manner, but Ecuador did not. The history of these two countries is evidence of the unpatriotic and immoral behavior recorded in the annals of the Latin American republics” (pp. 2-3).

The debt recorded at £1,424,579.05 rose to £2,393,293 by December 31, 1853 with interest at 6% per annum. New loans had to be renegotiated to cover the interest and overdue bonds. In some cases, these renegotiation agreements involved the use of vacant land as a form of payment, becoming an obstacle to economic development from the outset ( Liehr, 1989). During this republican stage, the country experienced a significant influence of military power and the church. The new republic was established on a poorly defined territorial order, with a network of interests, hegemony and domination at the national level, which led to the exclusion of the masses and the absence of a shared history among the indigenous and mestizos ( Acosta, 2006). Ecuador’s economy was framed in foreign trade, with cocoa as one of its main export products, which generated significant income through foreign exchange that contributed to the trade balance. This sector positioned itself as one of the main players in the global market, contributing to the generation of employment, economic growth and diversification of income sources. For Guerrero (1980) “this production boom gave rise to two social classes: cocoa and non-cocoa landowners, and bankers and traders, who were the main agents of the circulation of cocoa” (p. 82-83), called by Guerrero as the agro-financial and commercial oligarchy that achieved the complete integration of the production process and circulation of cocoa income (production chain); each member was a landowner, exporter and banker. This oligarchy was the result of the appropriation of large tracts of peasant land and the subjugation of its workers ( Chiriboga, 2013. p. 20-22), exploitation in which capital takes precedence over social aspects, resulting in underdevelopment. However, years later the situation was not the same; Spain being the main buyer of cocoa, this country limited the opening of the world market due to its refusal to recognize the independence of its colonies, which led to a decrease in exports, low prices and the abandonment of some plantations due to the decrease in income ( Maiguashca, 2012. p. 71-72).

Acosta (2006) the report describes at length Ecuador’s vulnerable economic condition as a result of the decoupling of cocoa production from the rest of the country’s economy, with surpluses going out as royalties on foreign investments, debt repayments and capital flight. In response to an economy in crisis, in 1843 the second Coin Law was issued giving way to the fractioning of the currency, twenty years later the Banco Particular de Guayaquil was authorized to issue currency and in 1895 the sucre entered into circulation as a measure to improve the economy. The problems of minting, banknote backing, depreciation, World War I and others increased the economic capacity of the agricultural and commercial bank until 1927 when the Central Bank (CB) was created. with the aim of ordering the monetary situation and controlling the stability of the exchange rate. The CB assumed this condition, although leaving aside other important economic aspects such as deflation (1928-1932) and inflation (1936-1937) ( Oleas, 1995, p. 99). The central bank reduced its involvement in macroeconomic aggregates and fiscal policy issues.

In 1940, the international market was opened for Ecuador, bananas stood out as the main product, and in 1946 there was a moderate growth in exports without reaching the same proportion as cocoa. Once again, the surpluses generated were invested outside the country, allowing exporters and producers to obtain large profits. These revenues were used to renegotiate the foreign debt, which by 1950 had increased to US$68.3 million. During this period, external financing for Latin America was consolidated, strengthening the support of the World Bank, the Inter-American Development Bank and the International Development Agency ( Acosta, 2006, p. 106). Competition in the Central American market increased, the spread of a plague affecting banana plantations allowed the expansion of international markets, consolidating Ecuador’s participation until 1955. Cocoa, bananas and rice continued to be the main agricultural export products ( Reyna, 2021, p.16); however, dependence on a monoculture economy, oriented to the export of primary products and with limited diversification conditions, led the country to a vulnerable situation in the face of competition and the subjugation and price control of the world market, a situation that placed it in a peripheral position ( Mero, Piedra & Galeano, 2017, p. 208).

**3.1.2 Oil as a powerful product to get out of the crisis**

Conditions such as political instability, constant changes of president, international competition, little national production, caused economic dependence on traditional export products that plunged into serious crises. It was necessary to consolidate production in other sectors in order to increase the State’s income, which led to an increase in indebtedness and dependence on international policies. In 1850, the first oil exploitation began in the coastal zone without favorable results and with a rather limited production that did not cover the internal demand, since production was less than the needs. In 1972, the exploitation process began in the eastern region of Ecuador. This period was marked by the colonization of land in the Amazon and the implementation of a development model with capitalist characteristics, driven mainly by the oil industry and the emergence of agro-industrial plantations ( Baca *et al*., 1997, p. 97). Oil extraction became a major activity, attracting investments and generating significant income for the country. At the same time, the expansion of agro-industrial plantations was encouraged, focusing on large-scale crops such as bananas, cocoa, oil palm, among others. This development model had a significant impact on the economy and the natural landscape of the Amazon region. For Rodríguez Lara oil revenues would help the problems of the marginalized people, who struggle in misery, ignorance and lack of health care ( Oleas, 1995). In effect, the exploitation and commercialization of oil would be the guarantee for the state’s income, whose main objective was to invest in social projects that would reduce the difficult conditions of the majority of the population. The state participated in a percentage of 63% of the total net income per barrel, although when taxes and royalties were added, 66% corresponded to the state, “this was the time in which it acted wisely and in accordance with the principles of the nation in defense of the interests of the state, the resources served for the common good and not for private interests” ( Gordillo, 2004, p. 51). Ecuador, like some Latin American nations, was immersed in unfavorable conditions, the rise in oil prices improved public sector revenues, but at the same time there was a growing expansion of public spending, on the other hand, financing from international banks and imports increased. In order for the economy to continue expanding without inflation, it was necessary to depend on foreign debt ( CEPAL, 1985).

Ecuador’s foreign debt has increased from US$ 24.5 million in 1950 to US$ 324.6 million in 1972, US$ 3,530.2 million in 1980, US$ 12,052.0 million in 1990, US$ 11,335.4 million in 2000 and US$ 46,974.3 million in October 2021 ( Banco Central del Ecuador, 2022).

### 3.2 Current status

The existing inequality between the countries of the North and those of the South and their characterization as developing countries has a long history. After World War II, the economic and financial catastrophe left Latin American countries at a disadvantage compared to the rest of the world, with the consequent stagnation of production, which prevented them from maintaining growth and development. The Gross Domestic Product (GDP) in Latin American countries generated from their production was not enough to meet the internal investment needs in the social field, especially in education, health and production variables, being necessary to resort to international support through financial institutions that generally determine the conditions for each country, and that in most cases affect the population with lower incomes, widening the inequality gap and increasing poverty.

The major international financiers: International Monetary Fund (IMF) and World Bank (WB), support all countries that require capital injection, their main objective is to provide economic assistance to countries that need and require external aid to achieve local stability, promote industrial development or initiate a process of socioeconomic reconstruction. Within its lending capacity, it supervises the economic policies of borrowers, offers technical assistance, analyzes the condition of each country and the economic outlook. Its policies are usually accompanied by a series of corrective measures, with the assurance that these policies are appropriate ( Cadenas, 2021), and implemented to ensure their payment.

Some economic analysts consider these organizations as double-edged weapons, stressing the care that must be taken for safe negotiations, as in 2010, 2013, 2014, 2015 and 2017 were the years where the greatest controversies occurred almost at the same time in Chile, Argentina, Uruguay and Paraguay, countries where the economic measures imposed by the governments as a guarantee for access to a loan from the IMF ended in protests and multiple disagreements by the citizens. The last three decades represent a process of financial reforms that have led to uncertainty and constant volatility, as well as slow growth, crises and stabilization policies. Monetarist economic policies have diminished the purchasing power of families and companies, there has been a destructuring of currency spaces, and there have been many attempts at monetary union ( Girón & Correa, 2008, p. 9).

According to the latest report of the International Development Bank (IDB) the total debt of Latin America and the Caribbean has reached 117%, governments will have to allocate between 46 and 55% of their GDP for public debt obligations depending on the conditions of each country, for the multilateral development banks provide long-term loans at competitive rates and financial instruments to cover risks ( Powell & Valencia, 2023).

Ecuador, with an economic condition leveraged on the primary production of cocoa, coffee and bananas; and since 1972 the oil that supported its economic growth in the early years of its, and which led to considerable improvements in education and health, however, its pace has been considerably slower, and in 1982 in several components, such as poverty, social inequality and employment, there has been a trend towards stagnation or deterioration ( Larrea, 2006, p. 63).

Like the rest of the Latin American countries, Ecuador has historically resorted to financial entities to meet its income deficit, with the consequent economic policies that have affected the entire population. In 2010, in governments considered to be leftist, this submission was rejected because they considered that they paralyzed development and subjected countries to their collateral conditions; it was not a fight for poverty but a rejection of the exploitation of the capitalist model ( Harnecker, 2010); However, in 2019, in view of the economic measure ordered by the government of President Lenin Moreno, which consisted of the withdrawal of fuel subsidies, the reaction of social groups led to a general strike as a result of the “Paquetazo”. To while in Chile, days of riots and protests began due to the increase in the price of public transport fares and the consequent need for financing.

This dependence on the external sector made the country vulnerable to the events of international markets; exports represent approximately 20% of the Gross Domestic Product (GDP) and the imports vary between 15% and 20% of GDP, the degree of openness of Ecuador’s economy, seen from the participation in foreign trade is relatively high compared to other non-oil countries ( BCE, 2022).

On the other hand, data from the Central Bank of Ecuador (BCE) indicate that public external debt was $41,495.6 million in 2019 (16.1% more than in 2018), representing 38.6% of GDP (5.4% more than in 2018); it also had an average year-on-year variation rate of 11.4% between 1950 and 2019. This debt covered 79% of total external debt in 2019 and registered a figure of $42,383.5 million through November 2020. In recent years, after the COVID 19 crisis, Ecuador obtained $514.1 million from the World Bank to support its economic reactivation, this financing contributed to the design of mechanisms for the expansion of social protection programs and the strengthening of cash transfer systems for the most vulnerable families. The Ministry of Economy and Finance has approved that the International Fund for Agricultural Development (FIDA) to finance $23.4 million for the Sustainable and Appropriate Development in Rural Territories project to be executed by the Ministry of Agriculture ( Ministrio de Economía y Finanzas, 2020), by 2022 the external debt closes at $63.732 million.

Another important aspect to consider is the technological condition of the countries, because although before globalization it was difficult to know the scientific progress in the northern countries, today that situation should change, the transfer of knowledge and technological progress through the results of research can reach in real time to all countries of the world, and should be used so that all follow the same line and the philosophy of the United Nations “that no one is left behind” is fulfilled, however, is another component that in Latin America and Ecuador is not met, the knowledge arrives at least 30 years later.

**3.2.1 Some macroeconomic indicators**

The credit condition that the country maintains with external organizations involves attention through economic policies for the payment of such credits; according to the law, no more than 40% of the GDP can be destined to external payments. The following table presents some macroeconomic indicators ( Table 1).

**Table 1.** Ecuador Macroeconomic indicators 2022 (as from BCE 2021-2022, WB 2022).

Indicators	Value
Nominal GDP (billions of dollars)	115.05
Current GDP per capita	6.391.3
GDP growth annual percentage	2.9
Urban unemployment rate	4.95
Public external debt (millions of dollars)	63.732
External debt as a percentage of GDP	39.9%
National poverty rate	27.7
National extreme poverty rate	10.5
Inflation, consumer prices % p.a.	3.5
Personal remittances received (% GDP)	4.1
Poverty ratio at $2.15 per day % of population	3.2
Total unemployment (% of total labor force. ILO model estimation)	3.8%

### 3.3 Development support

The term development has evolved over time, being conceptualized in different ways by different authors. Although it has traditionally been associated with economic growth, this perspective is limited. Amartya Sen proposes a broader vision, where development implies not only economic progress, but also political and social progress, focusing on enabling individuals to live with dignity and freedom ( Sen, 1998) During the 1990s, especially in Latin America, the different development models were criticized, the effectiveness of adjustment measures was questioned, and a structuralist approach to address economic and social inequalities was proposed ( Mujica Chirinos & Rincón, 2010), these inequalities were absorbed by international cooperation, engaging the external community in the search for integrative solutions, especially in countries with high international economic dependence and poorly functioning economies, as evidenced by the growing external debt and social neglect ( Duarte & González, 2014).

The cooperation of the larger in technology and innovation, towards the weaker allows the coexistence of two different worlds, but this cooperation should be focused on a real will for growth and development of the countries of the south but not out of remorse ( Pérez, 2015).

In this action, the measurement of development solely through GDP is criticized and it is advocated to consider multidimensional indicators that include aspects such as quality of life, human development, natural resources and environmental sustainability, in which the joint participation of the state, market and society in key areas such as education, poverty reduction and environmental protection, linked to sustainable development with equal opportunities, prevails ( Bidaurratzaga, Bosca, & Sánchez, 2021), to change the reality of the regions towards economic growth, but in a socially fairer way ( SánchezOro-Sánchez, 2006, p. 193), The contribution of support agencies should differentiate each state and apply policies according to its internal reality in terms of history, institutions and culture, since the application of the same policies in different territories is incorrect ( Adelman, 2002).

### 3.4 The IMF and WB development actors

**Figure 1.**
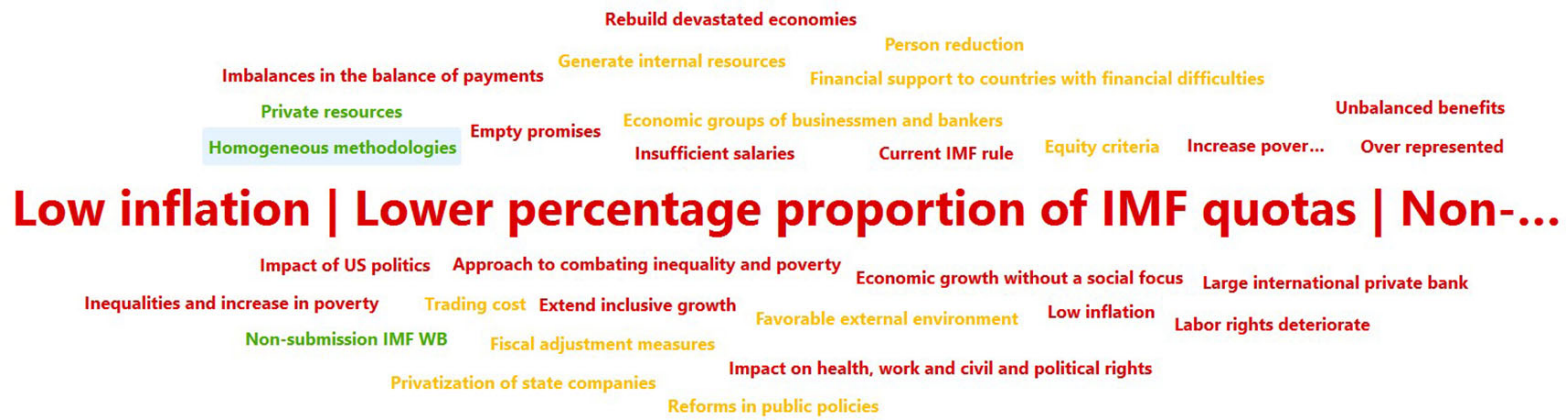
IMF – WB Development actors ( Romero et al., 2024).

The immediate purpose of the creation of the World Bank and the IMF was to promote economic development and full employment. The Figure 1 represens the times the concept is mentioned in the works analyzed for this article. After World War II, these institutions focused on restoring the monetary system and rebuilding damaged economies, however, the influence of private money limited their financing capacity and has led the IMF to impose adjustment measures on borrowing countries in times of crisis ( Arias & Vera 2002) and today the IMF’s ability to address financial problems and the inadequate representation of developing countries is questioned, since, the amount of loan that a member can request is not determined by its quota but by the improvement in 80-20 results, a rule that does not reflect the true demand for credit causing inefficiencies ( Miyakoshi, 2014).

The key challenge for the IMF and WB is to maintain inclusive growth and adapt to the new global economic order ( Sánchez, 2014), with negotiation and distribution costs low and assumed by the IMF ( Miyakoshi, 2014).

The World Bank (WB), initially the International Bank for Reconstruction and Development (IBRD) created to support the reconstruction of Europe and the financial stability of developing countries, has evolved in its lending mechanisms from an economic approach to a more social and sustainable one, from being just a bank to becoming a development agency facing crises and difficulties that have evidenced the failure of the initial hopes for the development of the economies of the region. Its new vision is aimed at “creating a world free of poverty on a livable planet”, actions are summarized in new variables such as energy, technology, food security, innovation, opening of trade flows, greater support to developing economies, especially those that maintain an average debt-to-GDP ratio of around 75%, with weak credit ratings in which borrowing costs are nine times higher than for better-rated economies. The latest proposals are aimed at joint actions between the IDB and the World Bank to make development effective ( Banco Mundial, 2023).

But, the promises of improving the welfare of the poorest have not been completely fulfilled, generating imbalances in the economic growth of developing countries ( Alacevich, 2010) (p.149). Several authors criticize the actions of these financing agencies, as they undermine labor rights and the standard of living of workers through policies that include downsizing, privatization of state enterprises, insufficient wages, wage inequality and social security violations, which benefit corporate interests to the detriment of the labor force ( Lloyd & Weissman, 2002) ( Arias & Vera, 2002).

The World Bank Group with more than 70 years of history, has adapted its policies in the international economy, impacting Latin America and promoting competitive and globalized market economies, prioritizing global capital over human rights ( Mendes, 2018). On the other hand, the IMF intervenes in economic crises assuming that the problems are similar (Casas, 2017), its lending policies, have consequences such as the lack of an established approach, subordination of the WB to the IMF, interaction with large private banks and the influence of the United States in these institutions, conditions that have led to an increase in the power of economic and financial sectors, reduction in the importance of the domestic market and a limited contribution to social ( Lichtensztejn, 2012).

These institutions have contributed to increasing inequality and poverty in the world, especially in Africa, with disastrous results in the 70’s and 80’s. Drajem (2001), Dembele (2005), Stubbs and Kentikelenis (2017) criticize the influence of these international financial institutions, by imposing austerity measures on countries with high levels of public debt, affecting human rights. Countries that did not follow IMF and WB recommendations during times of economic growth are considered to have been more successful in escaping poverty than those that did.

Attempts to redesign IMF policies to mitigate impacts on vulnerable populations have been insufficient, for Daoud, Herlitz, & Subramanian (2022), IMF policies have been criticized for not taking sufficient responsibility for the negative effects of austerity measures, increasing poverty in vulnerable countries.

Faced with these negative situations, the authors agree that these organizations should reform their institutional framework, so that poor countries participate in decision-making and achieve development objectives ( Alacevich, 2010, p. 149). Uncertainty should be considered and those affected should be listened to in order to design effective policies that promote freedom and autonomy (Casas, 2017). Propose reforms in public policies of self-sufficiency to help countries in economic crisis ( Daoud, Herlitz, & Subramanian, 2022), protect the health and standard of living of the population, develop equity metrics in applied studies and promote an economy focused on sustainable human development. A reorientation in these institutions is urgent to protect rights in education, health and work ( Stubbs & Kentikelenis, 2017).

The document prepared for the Latin American and Caribbean Regional Consultation on Financing for Development highlights the importance of a paradigm shift towards a sustainable development approach that considers social and environmental criteria, in addition to economic ones. It emphasizes the need to combine public financing with private resources to maximize development impact. The countries of the region, especially middle-income countries, should seek alternatives such as the mobilization of internal resources to achieve inclusive and sustainable development. It is crucial that they assume greater responsibility for their development and seek a favorable external environment that reduces asymmetries in the financial system, international trade and participation in technology and innovation; it is also important to consider opportunities for innovation, technology and knowledge transfer from developed countries ( CEPAL, 2015).

### 3.5 IMF and WB support to Ecuador

**Figure 2.**
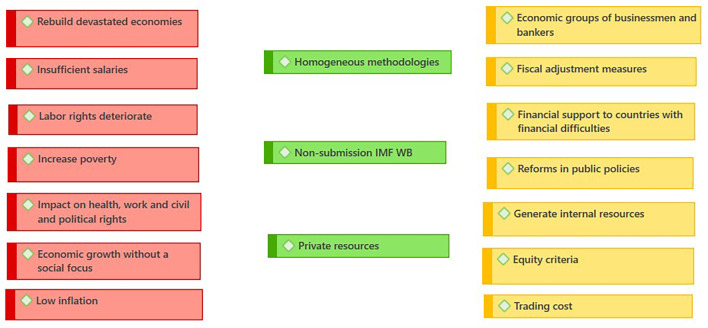
Consequences of WB - IMF credits. Ecuador ( Romero et al., 2024).

Expert analyses of the relationship between Ecuador and the IMF offer a multifaceted vision of the country’s economic and social dynamics. The Figure 2 represents the negative traits (red), possible improvements (yellow), positive factors (green); the following are the criteria of some authors:

Fernández and Santillana (2019) examine the letter of intent signed by Ecuador in 2019, highlighting the shift in the IMF’s discourse towards a focus on combating inequality and poverty, underlining the importance of the IMF’s constant monitoring to strengthen fiscal institutions and promote women’s participation in the labor market as generators of their own income. According to the latest report, 3,100 women-led small businesses receive financial support from the World Bank ( Figure 2) to strengthen women’s participation in the business sector ( Banco Mundial, 2023).

Contrasting this perspective, Weisbrot y Araúz (2019) criticize the IMF program and its effects on growth in Ecuador, pointing out that the proposed reforms may result in greater economic instability, lower GDP growth and an increase in unemployment and poverty. They highlight the accumulation of international reserves as a measure that, while increasing financial stability, does not necessarily translate into social benefits for the population.

In turn, García Shugulí (2020) examines the economic policies imposed by the IMF in the 1990s, arguing that these have contributed to increased poverty and migration, as well as decreased state investment in key areas such as education and health, this historical overview shows how the IMF’s structural adjustment policies have had negative repercussions on the Ecuadorian economy and society over time.

From a more contemporary perspective, Quise Llivipuma (2021) highlights the increase in public debt and the decrease in international reserves in Ecuador in recent years, pointing out the need for reforms to improve competitiveness in international markets, however, the conditions imposed by the IMF, such as the increase in VAT and fuel prices, pose challenges for the economic and social recovery of the country. This impact of the tax reforms imposed in the 2017-2021 period, which were implemented as measures to increase revenues and strengthen the economy were hampered by the pandemic and did not generate the expected results ( Garzón Falcón, 2022). Jepson (2022) also adds to these criteria with a complementary analysis of the Ecuador-IMF relationship, highlighting how China influence and political changes have disrupted domestic economic policies, negatively affecting the economy and income distribution ( Table 2).

**Table 2.** Analysis of IMF and WB development policies.

Author	Position with respect to financial institutions	Main ideas
Arias & Vera (2002)	Favorable position with reform proposal	Institutions conceived to create an international economic order based on Keynes theory. Conclusion: It is necessary to reformulate the IMF and WB to involve the participation of poor countries in the decision making process of territorial application, in order to achieve the development objectives ser by them.
Lloyd & Weissman (2002)	Favorable position with reform proposal	The role of IMF and WB policies in relation to workers in the labor force and labor law.
Sánchez (2004)	Favorable position	For the WB and the IMF, development was fundamental; they supported countres in economic difficulties through financial intervention. Criticism: In these organizations there is equal represenation of the majorty of countries.
Drajem (2001) y Dembele (2005)	Critical position	Institutions responsible for the reatest inequalities and the increase in poverty in the world, especially in Africa.
Rapkin, & Strand (2005)	Critical position on the quota systeem.	Developing countries are overrepresented when considering variables used by the IMF, if, for example, per capita GDP were taken, the results would be different.
De Souza (2009)	Position Critical of the idea of development	The USA will continue to dominate the world economy as the UN, IMF and WB are based in their states, there is a market position where the weaker must follow the stronger. The term development must be deconceptualized for a vision in which we are all different.
Alacevich (2010)	Critical position	The WB was created to support “economic” development, but later became a development agency for countries in crisis; improvements in welfare for all did not occur and the benefits of growth were more unbalanced.
Rowden (2010)	Critical position on policies	Need to create low inflationary levels for access to credit and need to strengthen inclusive development policies with educational and health equity. IFM programs have increassed health assistance in low-incorre countries.
Gupta (2010)	Positin in favor of health care	IFM programs have increased health care assistance in low-income countries.
Lichtensztejn (2012)	Critical position Diminishing opportunities for the domestic market.	They cause the monopolistic growth of strategic sectors led by businessmen and bankers and minimize the domestic market.
CEPAL (2015)	Critical position reduced participation in financing	Participation of countries in a favorable external environment and consideration of opportunities for scientific progress in developed countries.
Casas Julián (2017)	Critical position for similar recipes	The WB's initial objective was to reduce poverty, but poverty does not depend only on income; to overcome it, it is necessary to create freedoms and autonomy to develop sources of satisfaction and create their own uncertainty.
Stubbs & Kentikelenis (2017)	Critical position	WB and IMF demand policies that affect human, civil and political rights. A reorientation in the activities of these organizations is necessary.
Mendes (2018)	Critical position Mercantilist position diminishes Human Rights	Economic growth is moving from a hyper-mercantilist position to competitive and globalized markets, where the importance of human rights is diminished.
Daoud, Herlitz, & Subramanian (2022)	Critical position	Implementation of policies that affect the development of children, women and the elderly in particular. Austerity measures increase poverty
Neaime & Gaysset (2022)	Position in favor	Lack of international financing may hinder debt management and aggravate macroeconomic imbalances.

## 4. Discussion

Since the creation of the IMF and WB as financial organizations to overcome the post-war crisis, there have been several interventions in different countries, from a viewpoint of support to equalize their balance of payments and promote an international financial order, the contribution with the participation of the least favored countries has been quite limited in strengthening their development ( Arias y Vera, 2002), without equal representation ( Sánchez, 2014); The pandemic situation, the internal political crisis, and other macroeconomic factors cause poor debt management and increase macroeconomic imbalances ( Neaime y Gaysset, 2022).

The analysis of the authors’ criteria mostly coincides in pointing out that this financial support for Ecuador has generated greater indebtedness and dependence on the policies of these organizations throughout history, increasing the degree of inequality and exacerbating poverty, which especially affects children, women and the elderly ( Daoud, Herlitz y Subramanian, 2022). In recent years, the WB has become a development agency to support countries in crisis, therefore, according to the criteria of the analyzed authors, it is necessary to focus on sustainable projects that are framed in a sustained growth of income and support the achievement of rights, especially in education as a factor enabling the development of human capital ( Wang & Zheng, 2024, p. 19).

## 5. Conclusions

The historical condition of indebtedness of Ecuador since it was part of the Gran Colombia has been increasing over the years, several investigations from a historical approach determine the productive wealth of the country, as well as the commercial positioning of different products in international markets that, according to conditions such as the cyclical behavior of the economy, the emergence of competing products and the wrong decisions of the rulers did not allow since the early years of the Republic to settle that debt and in recent years has grown to exaggerated levels.

The studies examined in relation to development maintain that the demand for growth, prosperity and progress in various regions are fundamental to generate greater income in a country. These demands are predominantly evaluated from an economic perspective using indicators such as GDP, trade balance, monetary reserve, among others. However, in a capitalist system, terms such as improved living conditions, poverty reduction, education and health are often not given the same importance.

In relation to the support of international financial organizations for development in the context of Ecuador, the authors analyzed agree in the perception that the policies implemented by these organizations have reduced the opportunities for growth towards development, it is considered that these institutions act in a restrictive manner, imposing on debtor countries the adoption of internal policies that harm the most vulnerable sectors, which intensifies inequality and exacerbates poverty rates. Ecuador is currently facing the worst political and economic crisis in its history, a country devastated by drug trafficking, corruption and organized crime, which makes it highly vulnerable to a financial meltdown, the consequent need for external financing and therefore absolute dependence on the conditions of its financiers; in the medium term it will exacerbate inequality and poverty.

## Ethics and consent

Ethical approval and consent not required.

## Data Availability

No data are associated with this article. Zenodo: Chronological approach to the characteristics of Ecuador’s debt with the imf and the wb. https://doi.org/10.5281/zenodo.13131334 ( Romero *et al.*, 2024). The project contains the following files:
•
Figure 1 •
Figure 2 •
Table 1 •
Table 2 Data are available under the terms of the Creative Commons Attribution 4.0 International license (CC-BY 4.0).

## Software availability

- Atlas’ti ( https://atlasti.com/es)
- MethodologyPRISMA ( https://biblioguias.unav.edu/revisionessistematicas/guias_oficiales)
