# Supplementary material for: Chronological approach to the characteristics of Ecuador’s debt with the IMF and the WB
Source: F1000Res. 2025 Jan 23;13:957. Originally published 2024 Aug 23. [Version 2] doi: 10.12688/f1000research.154378.2 (PMC11791397; doi:10.12688/f1000research.154378.2)
Supplement: Peer review history

# Reviewer report: Reviewer response for version 2

**DOI:** 10.5256/f1000research.177139.r361914

**Published:** 2025-02-03

**Authors:** Halder Yandry Loor Zambrano

**Affiliations:** 1 Universidad Tecnica de Manabi, Portoviejo, Manabí Province, Ecuador

The author has taken positively the recommendations made by the reviewer, we are pleased to contribute to the scientific enrichment of the proposed document, in which some statistical data from official information sources (Central Bank of Ecuador) have been added, concerning the oil and agricultural production of this country, which is useful for future readers and researchers, who will be nourished with relevant updated information on this topic.  Likewise, bibliographic references were incorporated in the discussion section, with criteria from different points of view for the debate, between authors in favor and those who are against the participation of multilateral international credit organizations in the financing of Ecuadorian development projects and plans, which contributes significantly to the analysis, since it shows a justified balance, in a responsible manner, in the conclusions of the case study, without political or ideological bias, which is, after all, what a document of this nature should pursue.

Is the case presented with sufficient detail to be useful for teaching or other practitioners?

Partly

Is the work clearly and accurately presented and does it cite the current literature?

Partly

If applicable, is the statistical analysis and its interpretation appropriate?

Yes

Are all the source data underlying the results available to ensure full reproducibility?

Yes

Are the conclusions drawn adequately supported by the results?

Partly

Is the background of the case’s history and progression described in sufficient detail?

Yes

Reviewer Expertise:

Finance, business plan, project management, management,

I confirm that I have read this submission and believe that I have an appropriate level of expertise to confirm that it is of an acceptable scientific standard.

# Reviewer report: Reviewer response for version 2

**DOI:** 10.5256/f1000research.177139.r361915

**Published:** 2025-01-24

**Authors:** Ramón García Marín

**Affiliations:** 1 University of Murcia, Murcia, Region of Murcia, Spain

The authors have made the changes suggested by the reviewers, so this article has been substantially improved. I am glad to be able to contribute to the indexing of excellent papers. Congratulations!

Is the case presented with sufficient detail to be useful for teaching or other practitioners?

Yes

Is the work clearly and accurately presented and does it cite the current literature?

Yes

If applicable, is the statistical analysis and its interpretation appropriate?

Yes

Are all the source data underlying the results available to ensure full reproducibility?

Yes

Are the conclusions drawn adequately supported by the results?

Yes

Is the background of the case’s history and progression described in sufficient detail?

Yes

Reviewer Expertise:

Human Geography; Economic Geography

I confirm that I have read this submission and believe that I have an appropriate level of expertise to confirm that it is of an acceptable scientific standard.

# Reviewer report: Reviewer response for version 1

**DOI:** 10.5256/f1000research.169394.r333697

**Published:** 2024-12-31

**Authors:** Halder Yandry Loor Zambrano

**Affiliations:** 1 Universidad Tecnica de Manabi, Portoviejo, Manabí Province, Ecuador

The case study is extremely interesting and presents relevant data and the PRISMA methodology is appropriate for this type of work.

 However, I consider that they should update the study at the level of agricultural and oil production and some macroeconomic indicators of Ecuador, which may well be provided by the Central Bank of Ecuador, since we are in the year 2024.

 Likewise, although the criteria of the different authors cited in this document mostly coincide in pointing out that financial support for Ecuador has generated greater indebtedness and dependence on the policies of these organizations throughout history, I consider that they should also be included.

 in this case study, especially in the discussion section, to later refer to in the conclusions (to complete the analysis), those criteria that do not share this argument, given that, being multilateral credit organizations, they must ensure because that financial resource that they grant via loans to countries that request them, return to their coffers. It must be remembered that these institutions do not oblige any country to request this type of financial requirements and they are not responsible for the fact that certain rulers in power, in some countries and in the case of Ecuador specifically, have allocated the funds for an excessive increase in bureaucracy. waste of financial resources, corruption, among other evils (endemic in some South American countries), forgetting the origin of this type of funds, which must be returned according to the established deadlines and conditions.

Is the case presented with sufficient detail to be useful for teaching or other practitioners?

Partly

Is the work clearly and accurately presented and does it cite the current literature?

Partly

If applicable, is the statistical analysis and its interpretation appropriate?

Yes

Are all the source data underlying the results available to ensure full reproducibility?

Yes

Are the conclusions drawn adequately supported by the results?

Partly

Is the background of the case’s history and progression described in sufficient detail?

Yes

Reviewer Expertise:

Finance, business plan, project management, management,

I confirm that I have read this submission and believe that I have an appropriate level of expertise to confirm that it is of an acceptable scientific standard, however I have significant reservations, as outlined above.

# Response

**Published:** 2025-01-09

**Authors:** Martha Lucía Romero Flores

**Affiliations:** Facultad de Ciencias Políticas, Universidad Nacional de Chimborazo, Riobamba, Chimborazo Province, Ecuador

Dear Reviewer

 Thank you for your comments, which have been accepted and adjusted based on the recommendations and inserted in the text.

 I remain attentive to the indications for the respective correction.

 Kind regards

# Reviewer report: Reviewer response for version 1

**DOI:** 10.5256/f1000research.169394.r320035

**Published:** 2024-10-10

**Authors:** Ramón García Marín

**Affiliations:** 1 University of Murcia, Murcia, Region of Murcia, Spain

Ecuador's foreign debt has been a constant concern throughout the country's history, ever since it gained independence in the early 19th century. Bad loans and poor negotiations increased the foreign debt, which was in many cases the result of corruption, as very important resources were diverted for private purposes. The basic needs of the Ecuadorian population, such as health, education, housing and other infrastructure necessary for its development, have been neglected. 

 This research, from my point of view, is original, timely and necessary.

 The paper clearly contextualizes the problem and justifies the choice of the topic of analysis. The research questions, objectives and hypotheses are clear and well defined.

 Even though it is brief, there is a good and clear description of the research design (Analysis Methodology).

 Regarding the Results, the authors present the relevant information; they are well organized and supported with graphs and tables. The conclusions offer an overview of the main findings and a well-posed general assessment is presented.

 Regarding writing, style and format, the writing is fluid, clear, concise and understandable. The grammar and spelling are correct. There are no errors. There are clear transitions between chapters, subsections and paragraphs. And the citation format in the text and bibliography is applied correctly throughout the document.

 Therefore, and to conclude, I believe that the indexing of this research is appropriate.

Is the case presented with sufficient detail to be useful for teaching or other practitioners?

Yes

Is the work clearly and accurately presented and does it cite the current literature?

Yes

If applicable, is the statistical analysis and its interpretation appropriate?

Yes

Are all the source data underlying the results available to ensure full reproducibility?

Yes

Are the conclusions drawn adequately supported by the results?

Yes

Is the background of the case’s history and progression described in sufficient detail?

Yes

Reviewer Expertise:

Human Geography; Economic Geography

I confirm that I have read this submission and believe that I have an appropriate level of expertise to confirm that it is of an acceptable scientific standard.

# Response

**Published:** 2024-10-12

**Authors:** Martha Lucía Romero Flores

**Affiliations:** Facultad de Ciencias Políticas, Universidad Nacional de Chimborazo, Riobamba, Chimborazo Province, Ecuador

Dear Doctor

 I would like to thank you for your approval of the article:

 Chronological approach to the characteristics of Ecuador's debt with the IMF and the WB, which reveals from a historical approach, the situation of risk that Ecuador lived and that in the present time has transformed its external debt into eternal and exponential debt. This article will serve as a support for researchers in the economic and social field.
